# *Histoplasma capsulatum* prosthetic valve endocarditis treated with oral isavuconazole

**DOI:** 10.1016/j.mmcr.2025.100723

**Published:** 2025-08-09

**Authors:** Colton Boney, Alyssa Zakala, Aimee League, Ali Hassoun

**Affiliations:** a The Alabama College of Osteopathic Medicine, 445 Health Sciences Blvd, Dothan, AL, 36303, USA; b Huntsville Hospital, 101 Sivley Rd SW, Huntsville, AL, 35801, USA; c The University of Alabama School of Medicine, Huntsville Hospital, 301 Governors Dr SW, Huntsville, AL, 35801, USA

**Keywords:** Histoplasmosis, Fungal endocarditis, Endemic mycoses, Isavuconazole, *Histoplasma capsulatum*

## Abstract

*Histoplasma capsulatum* remains the most common cause of systemic fungal infections in the United States, with severe cases occurring in older adults, immunosuppressed individuals, and those with lung disease. Despite its prevalence in systemic and pulmonary illness, histoplasmosis endocarditis is an unlikely source of mycotic endocarditis, with limited updated guidelines on antifungal therapy. We present a case of artificial valve *histoplasma* endocarditis requiring surgery and antifungal therapy. Isavuconazole was selected through shared decision-making due to concerns about the tolerability of traditional agents. The patient was successfully treated with a combination of aortic valve replacement and isavuconazole therapy.

## 1 Introduction

*Histoplasma capsulatum* is a dimorphic fungus found on most continents, excluding Antarctica. Worldwide, it is a leading cause of systemic fungal infections. In the United States, this organism is most prevalent in the Ohio River Valley, however it has been reported well outside this region [1]. *Histoplasma* thrives in decaying organic material and bird or bat droppings, making agriculture, construction, and outdoor activities high-risk exposures. Though most infections are typically asymptomatic, around 5 % of cases present with self-limiting respiratory symptoms. Less commonly, there are documented severe cases occurring in individuals who are elderly, immunocompromised, or have underlying lung disease [1]. Histoplasmosis can also spread from an original site, most commonly the lungs, to other organ systems in what's known as disseminated histoplasmosis. Disseminated histoplasmosis primarily affects the liver, adrenal glands, and spleen [1].

Mycotic endocarditis is an uncommon type of endocarditis, usually attributed to Candida and Aspergillus species [2]. Histoplasmosis endocarditis is an exceedingly rare source of mycotic endocarditis, with limited data on treatment outcomes using newer antifungals.

The Infectious Diseases Society of America (IDSA) recommends liposomal amphotericin B followed by itraconazole for at least one year for disseminated histoplasmosis [3].

Mild-to-moderate cases may be treated with itraconazole alone [3]. We present a case of bioprosthetic aortic valve *Histoplasma* endocarditis successfully treated with surgical intervention and antifungal therapy. Due to concerns over traditional antifungals, the patient was treated with isavuconazole, resulting in a sustained clinical response without complications.

## 2 Case

A 76-year-old male presented to the emergency department on day 25 of fever, chills, and weight loss. Upon further investigation, the patient additionally reported gradual worsening of fatigue and dyspnea over one year duration. His significant past medical history includes severe coronary artery disease with prior myocardial infarction needing coronary artery bypass grafting, transcatheter aortic valve replacement with bioprosthetic valve (TAVR), and pseudogout with recent prednisone taper. During the interim, patient was receiving iron transfusions for his iron deficiency anemia as well as anticoagulation adjustments for bioprosthetic aortic valve degradation as seen on outpatient echocardiogram. He originally attributed his new symptoms to ongoing medical conditions, however severity of symptom progression with development of intermittent fevers on day 0 prompted him to seek medical care after three and a half weeks without improvement.

**Fig. 1:**
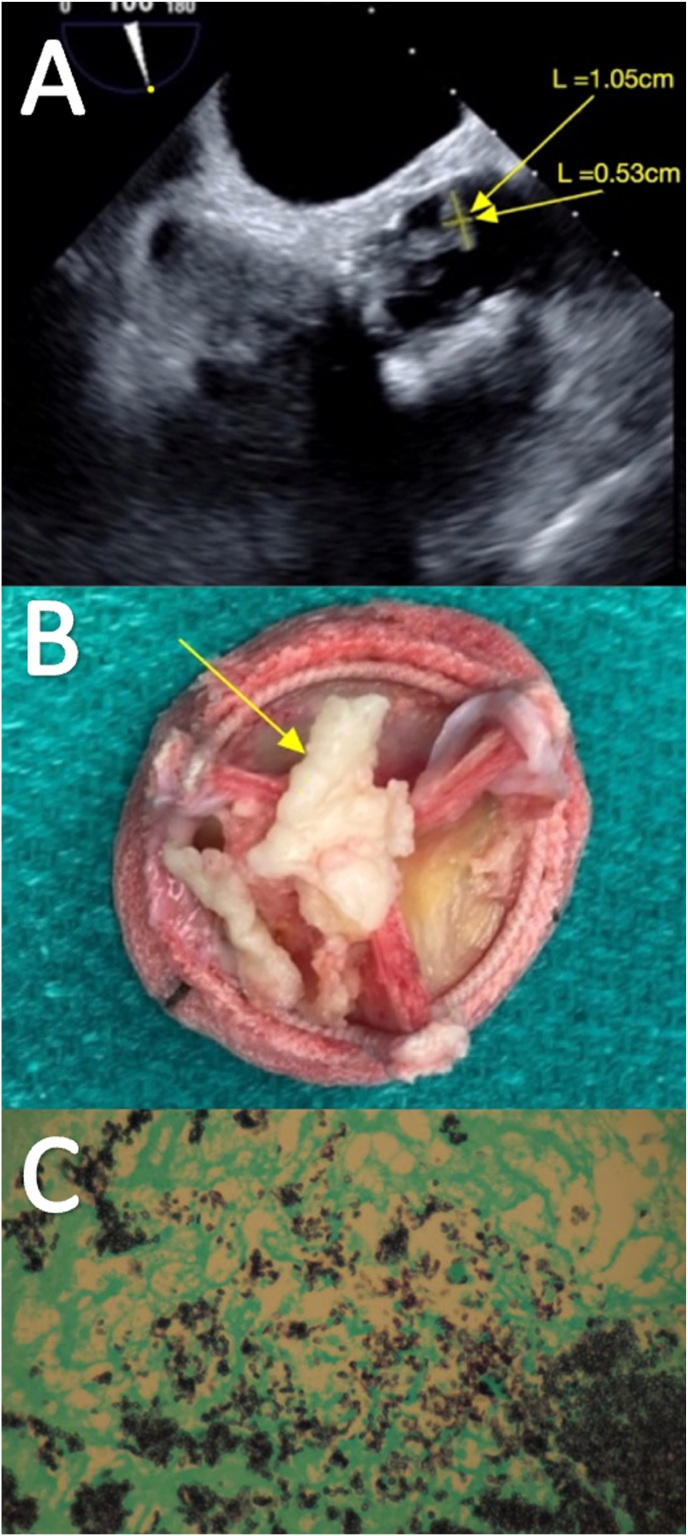
(**A**) Transesophageal echocardiogram performed at hospital admission showing a hypermobile mass on the bioprosthetic aortic leaflet measuring 1.05x0.53cm. (**B**) Diseased prosthetic aortic removed by open valve replacement. Arrow depicts the large white mass previously seen on TEE that was sent to pathology. (**C**) Grocott's methenamine silver stain of the valvular mass showing small black oval infiltrates seen diffusely throughout degenerative debris of the fibrin clot.

A repeat transthoracic echocardiogram identified a new aortic valve mass, leading to hospitalization. Admission labs revealed normocytic anemia (hemoglobin 10.4 g/dL, hematocrit 32.2 %), elevated C-reactive protein (CRP) (4.6mg/dL), and metabolic acidosis. Blood cultures and HIV testing were negative. A transesophageal echocardiogram was ordered to further characterize his new aortic mass, showing a highly mobile 1.05 cm × 0.5 cm echodensity (image A, see Fig. 1) on the prosthetic aortic valve. The cardiothoracic surgical team was consulted, and based on the patient's high embolic risk, the patient ultimately underwent an open aortic valve replacement. The excised valve (image B), was sent to pathology, stained with Grocott's methenamine silver, showed fungal elements throughout the mass (Image C). Urine antigen and complement fixation tests were positive for *Histoplasma capsulatum*, supporting the diagnosis. Additionally, fungal blood cultures were obtained using Sabouraud agar. After 15 days, fungal blood cultures grew septate-hyphae mold with spherical, tuberculate macroconidia and PCR of this sample confirmed the diagnosis of *Histoplasma capsulatum* aortic valve endocarditis.

Following the surgery, infectious disease recommended long-term antifungal therapy. Through shared decision-making, the patient expressed concerns about adverse side effects with the use of liposomal amphotericin B, as well as adherence difficulty with itraconazole's frequent monitoring requirements. Oral isavuconazole was ultimately selected and administered at a loading dose of 372mg every 8 h for 48 h followed by 372 mg once daily as maintenance for a goal of 12 months of therapy Although not FDA-approved for histoplasmosis, isavuconazole has been used off-label for pulmonary and disseminated infections due to its favorable safety profile and bioavailability.

After nine months on antifungal therapy, he remains asymptomatic with normalization of CRP, negative urine antigen, and reduced complement fixation titers (from 1:512 to 1:32). Echocardiography confirmed stability of the new prosthetic valve. Treatment is planned for a total of 12 months with reevaluation of extending antifungal therapy.

## 3 Discussion

Endocarditis is most often caused by bacteria, however negative blood cultures with constitutional symptoms and new echocardiographic abnormalities should prompt investigation for endocarditis from an atypical source [2]. If there is suspicion for endocarditis, fungal causes should be included in the differential for immunocompromised individuals or those with prosthetic valves given the increased risk of fungal seeding. Fungal blood cultures are the diagnostic gold standard for fungal endocarditis [2]. However, the long result times for cultures make additional diagnostics, such as [[1], [2], [3]]-β-d-glucan, galactomannan assays and polymerase chain reaction tests, valuable in ruling out *Candida*, *Aspergillus,* and other fungal pathogens. Emerging techniques like cell-free DNA sequencing may improve pathogen detection and guide therapy, but preliminary data on their use in culture negative endocarditis is limited [4].

There are many testing modalities for histoplasmosis. Diagnosing histoplasmosis often involves urine antigen detection and complement fixation or immunodiffusion antibody tests. These rapid, noninvasive tests are crucial in critically ill patients [5]. Histopathology aids in distinguishing *Histoplasma* from conditions like sarcoidosis or malignancy [1]. Fungal cultures, while definitive, have variable sensitivity and take weeks to finalize. Despite an associated time delay for results, they are necessary for infectious species identification as there is cross-reactivity of *Histoplasma* and *Blastomyces* in urine antigen testing [6].

*Histoplasma* endocarditis is rare, with only 25 prosthetic valve cases in the literature between 1940 and 2020 [7]. Cases were equally distributed between native and prosthetic valves, predominantly affecting men. Complement fixation was the most sensitive diagnostic method with positive results in 13/15 cases, while fungal cultures from non-cardiac sites were negative in half of cases [7].). Site selection for fungal cultures seem to play an important role as cultures from non-cardiac sites were negative in half of cases and cultures from cardiac valve emboli were positive in all cases [7]. Without intervention, mortality was 100 % and antifungal therapy alone had a 50 % mortality rate. The best outcomes were seen with combined surgical and antifungal treatment [7].

Therapeutic options for histoplasmosis endocarditis are limited. IDSA guidelines for histoplasmosis treatment stratify management based on disease severity and CNS involvement [3]. Mild-to-moderate disseminated histoplasmosis is treated with itraconazole for one year, while severe cases require liposomal amphotericin B followed by itraconazole [3]. The American Society for Microbiology (ASM) 2023 fungal endocarditis review recommends liposomal amphotericin B as the primary therapy [2]. The ASM guidelines include alternative regimens for many fungal pathogens that may cause endocarditis, but none are listed for endemic mycoses like *Histoplasma* due to limited data.

Of the endemic mycoses, *Histoplasma* is the most common, yet there are fewer than 100 documented endocarditis cases [7]. Many cases predate the introduction of newer triazoles like posaconazole and isavuconazole. Isavuconazole offers many advantages compared to itraconazole. Isavuconazole offers an improved safety profile compared to itraconazole, including reduced hepatotoxicity and no QTc prolongation [8,9]. Likewise, isavuconazole does not require therapeutic drug monitoring given its higher bioavailability [9]. Further amplifying the bioavailability difference, itraconazole is recommended to be taken with acidic beverages to ensure maximum drug absorption [9]. Isavuconazole's absorption is not altered by gastric pH [9]. While side effects such as nausea, diarrhea, and transaminitis can occur with either agent, the frequency of these events is significantly higher in itraconazole use [9].

Only one other case has documented successful isavuconazole use in *Histoplasma* endocarditis, after liposomal amphotericin B and itraconazole were discontinued due to adverse effects and subtherapeutic levels, respectively [10]. These challenges (poor tolerance of liposomal amphotericin B, itraconazole's drug interactions, and its requirement for dietary intake and therapeutic monitoring—highlight the need for alternative therapeutic options. While fluconazole can be used as step-down therapy, it has been associated with treatment failure and relapse [11]. There are limited cases in the literature where isavuconazole has been used successfully for disseminated histoplasmosis without cardiac involvement [10,[12], [13], [14]]. Further research is needed to evaluate newer triazoles for mild-to-moderate disseminated histoplasmosis and as step-down therapy following amphotericin B. This case adds to the limited literature of isavuconazole being used as alternative therapy for disseminated histoplasmosis endocarditis. More data is needed on the efficacy of newer triazole antifungals in the treatment of histoplasmosis.

## CRediT authorship contribution statement

**Colton Boney:** Writing – original draft, Visualization, Investigation, Conceptualization. **Alyssa Zakala:** Writing – review & editing, Visualization, Investigation. **Aimee League:** Visualization, Supervision, Project administration, Investigation. **Ali Hassoun:** Writing – review & editing, Supervision, Resources, Investigation.

## Conflict of interest

There are none.
